# Spectroscopic investigation of faeces with surface-enhanced Raman scattering: a case study with coeliac patients on gluten-free diet

**DOI:** 10.1007/s00216-022-03975-y

**Published:** 2022-03-08

**Authors:** Stefano Fornasaro, Alessandro Esposito, Fiorella Florian, Alberto Pallavicini, Luigina De Leo, Tarcisio Not, Cristina Lagatolla, Marica Mezzarobba, Alessia Di Silvestre, Valter Sergo, Alois Bonifacio

**Affiliations:** 1 Raman Spectroscopy Laboratory, Department of Engineering and Architecture, University of Trieste, P.le Europa 1, 34100 Trieste, Italy; 2 Department of Life Sciences, University of Trieste, Via Edoardo Weiss 2, 34128 Trieste, TS Italy; 3 Institute for Maternal Child Health-IRCCS “Burlo Garofolo” Trieste, via dell’Istria 65/1, 34100 Trieste, Italy

**Keywords:** Faeces, SERS, Nanoparticles, Raman, Gold

## Abstract

Surface-enhanced Raman scattering (SERS) spectra of faecal samples can be obtained by adding AuNP to their methanol extracts according to the reported protocol, and display bands that are due to bilirubin-like species but also to xanthine and hypoxanthine, two metabolic products secreted by gut bacteria. A total of 27 faecal samples from three different groups, i.e. coeliac patients (*n* = 9), coeliac patients on gluten-free diet (*n* = 10) and a control group (*n* = 8), were characterized with both SERS spectroscopy and 16S rRNA sequencing analysis. Significant differences are present between SERS spectra of coeliac patients and those on gluten-free diet, with a marked increase in the relative intensity of both xanthine and hypoxanthine for the latter. Interestingly, these differences do not correlate with bacterial composition as derived from 16S rRNA sequencing.

**Supplementary Information:**

The online version contains supplementary material available at 10.1007/s00216-022-03975-y.

## Introduction

Metabolomics is a systems biology approach that aims at the qualitative and quantitative analysis of small molecules (below 1.5 kDa) in biological samples. The most frequently explored specimens for metabolomic analyses in humans are serum, urine and tissues. More recently, faecal samples have become a valuable choice, since they can be obtained noninvasively. Human faeces are a very complex biological matrix with a broad biochemical composition that represents a rich source of diverse metabolic compounds derived from the host, the gut microbiota and xenobiotics. Over the last decade, with the rapid development of microbiome sequencing technologies, an integrated omics analysis of faecal material has gained attention as a non-invasive method for studying the complex interactions between the human gut microbiota (GM) and the host [1]. The human GM is characterized by diverse microbial communities of multiple phyla of bacteria, archaea, viruses and microbial eukaryotes [2], which perform many essential protective, structural and metabolic functions for human health, including food processing, digestion of complex indigestible fibres, pathogen displacement and synthesis of many compounds responsible for regulating the activity of distal organs (e.g. the brain [3]). The influence of the GM in regulating metabolic activity is now recognized with increasing evidence. For instance, the faecal microbiome and metabolome are simultaneously found to be disordered in colorectal cancer [4], systemic lupus erythematosus [5], metabolic syndrome [6], asthma [7] and central nervous system disorders [8]. In these settings, the faecal metabolic profiles complement sequencing-based approaches to provide a functional readout of the gut microbiome and gain insight for improving the diagnosis and prognosis of several diseases. The most frequent approach to study the microbiota composition is to target the bacterial 16S ribosomal RNA (rRNA) extracted from faecal samples [9]. However, this approach is typically laborious and expensive for application on small batches of samples, as it is common in clinical practice; moreover, factors that influence microbial RNA stability can produce a significant variation in the gut microbiome composition. On the other hand, the current state-of-the-art technology in faecal metabolomics is the hyphenation of high-resolution mass spectrometry–based techniques (HRMS) with high-performance chromatography. HRMS is a universal detection technique that presents a very high selectivity and sensitivity offering simultaneous structural and quantitative information. However, quantitative information can be only gathered via the analysis of calibration standards for the compounds of interest. More importantly, HRMS–based techniques are expensive and time consuming and require specialized personnel, making them unsuitable for many health-care routines. Thus, new versatile, cost-effective and fast alternatives for the accurate identification of faecal metabolites, rapid acquisition and detection methods are needed.

Surface-enhanced Raman scattering (SERS) spectroscopy is an emerging analytical technique for metabolomic analysis applied to clinical needs, due to its non-destructive nature and single-molecule detection ability [10, 11]. The amplification of the inelastic scattering of light by molecules in close proximity to electromagnetic “hotspots” on the plasmonic nanostructures (i.e. the SERS substrates) allows for the transduction of compounds at low concentration to measurable spectral signals. SERS spectra can be easily obtained from solutions by minimally trained personnel, with relatively inexpensive instrumentation and without complex sample preparation. In label-free SERS, each spectrum contains information about the molecules that freely adsorb on the substrate’s surface. SERS spectra depend on the relative concentration of the metabolites present in a biofluid as well as on their chemical affinity for the substrate’s surface, the latter factor being the most relevant. Containing information mainly due to low-molecular-weight metabolites, label-free SERS spectra of biofluids provide a “biochemical snapshot” of potentially clinically relevant information about the metabolic status of a subject, especially in cases where little is known about the biomolecular species responsible for the studied condition.

Therefore, the aim of the present study was to develop a fast and sensitive method that yields reliable SERS spectra from human faeces, and characterize them in terms of the amount/quality of information obtainable from that specific matrix, using a simple protocol and a compact and portable instrument. Additionally, as a case study, we applied the developed SERS method to faecal samples collected at diagnosis from paediatric patients with coeliac disease (CD) following a gluten-containing diet as well as from coeliac patients following a gluten-free diet (GFD). CD is an autoimmune condition, secondary to an immunological response to ingested gluten, in genetically susceptible individuals. Once the diagnosis is achieved, the only existing treatment is a lifelong GFD. Clinical manifestations of untreated CD, such as anaemia, depression, infertility and osteoporosis can improve with a GFD [12]. Thus, strict adherence to a GFD is critical to reduce symptoms, avoid nutritional deficiencies and increase quality of life. Moreover, GFD compliance should be monitored to avoid cumulative damage. Although not yet exhaustive, the current literature suggests an association between CD, gut microbiota and the metabolome [13–15].

To the best of our knowledge, this is the first SERS study on the faecal profiling of the biochemical perturbations that accompany coeliac children under GFD.

## Experimental section

### Reagents

Methanol used to obtain faecal extracts, hypoxanthine, xanthine and bilirubin, and all the chemicals and solvents used in the synthesis of AuNP were purchased from Merck (Merck KGaA, Darmstadt, Germany). E.Z.N.A® Stool DNA kit (Omega Bio-Tek) was used to extract stool DNA. AccuStartII PCR ToughMix 2X (Quanta Bio) plus EvaGreen™ 20X (Biotium) was used in real-time amplification. Mag-Bind®TotalPure NGS (Omega Bio-Tek) was used to purify PCR products. A Qubit dsDNA HS Assay Kit (Thermo Fisher Scientific) was used to quantify PCR products. Ion PGM™ Template Hi-Q OT2 400 View, Ion PGM™ Enrichment Beads and Ion PGM™ Hi-Q™ view Sequencing Kit (Thermo Fisher Scientific) were used for sequencing.

### Faecal samples

Samples were collected by IRCCS Burlo Garofolo from subjects instructed to collect and keep a sample of stools at − 20 °C. Sample characteristics are reported in Table 1 and in Supplementary Fig. S1. Specimens were delivered and kept at − 20 °C until analysis. Once thawed, the samples were homogenized and two aliquots were prepared for SERS and genomic analysis. Written informed consent was obtained from the parents of the children enrolled, and the study was approved by the hospital’s independent ethical committee (CEUR-2019-Os-157). Samples from CD patients were collected at the time of diagnosis, and from patients in GFD from at least 1 year.

**Table 1:** Number, sex and age characteristics of the stool sample donors enrolled in the study. For details, see Supplementary Fig. S1

	F	M	Total	Median age (quartiles)
CTR	3	5	8	7 (5.75–10)
CD	7	2	9	10 (4–14)
GFD	7	3	10	12.5 (12.0–15.5)
Total	17	10	27	

### SERS substrates

The aqueous colloidal dispersion of gold nanoparticles (AuNP) used as SERS substrates was synthesized according to the method of Turkevich et al. [16], involving the reduction of Au(III) salts with sodium citrate. All solutions were prepared with ultrapure water, MilliQ (Millipore, USA), and all the glassware was cleaned with a Nochromix® (Godax Laboratories, Inc.) solution (with H_2_SO_4_), *aqua regia* (1 HNO_3_:3 HCl, vol.), and finally thoroughly rinsed with MilliQ water before use. Operatively, 10.6 mg of NaAuCl_4_·2H_2_O (sodium tetrachloroaurate dihydrate) was added to 25 mL of water in an Erlenmeyer flask and heated to boiling. Then, 750 μL of sodium citrate tribasic dihydrate aqueous solution (1%, 1 g/100 mL) was rapidly added, and the solution was kept boiling for 20 min under vigorous stirring and reflux using a water-cooled condenser. Ultimately, the colloidal dispersion was left to cool down to room temperature. Nanoparticles were characterized by UV–visible spectroscopy (Cary100, Agilent, Santa Clara, USA) and transmission electron microscopy (EM 208, Philips, Amsterdam), and had an average/median size of 53.6 nm. The UV–visible extinction spectrum, TEM micrograph and size distribution (as calculated from TEM images) are reported in Supplementary Information (Fig. S2).

### SERS measurements

Aliquots of 125 mg of faeces were dispersed in 5 mL of methanol and vortexed for 30 s to obtain a methanol faecal extract. Twenty-microlitre aliquots of this faecal extract were micropipetted into 1.5-mL Eppendorf tubes containing 180 µL of the colloidal dispersion of AuNP, and mixed by repetitive pipetting. Fifty microlitres of these mixtures was then deposited as a drop onto a CaF_2_ microscope slide, ready to be measured by SERS. Methanol solutions of bilirubin (20 µM), hypoxanthine (10 µM) and xanthine (20 µM) were prepared by a direct dilution of stock solutions in aqueous NaOH (0.1 M for bilirubin, 1 M for xanthine and hypoxanthine) with methanol. For SERS measurements, AuNP were added to a 1:9 ratio to the methanol solutions of these metabolites (following the same protocol for faecal extracts). The spectra collection was performed in air at room temperature with an i-Raman Plus portable system (BWS465-785S) through a compatible Raman video microscope (BAC151B) and with the BWSpec software (version 4.03_23_c), by B&W Tek (Newark, DE). Excitation was obtained with a 785-nm laser with an output power of about 400 mW. Laser light delivery to the sample and scattering collection occurred through an optical fibre probe connected to a compatible Raman video microscope. The instrument spectrograph had an average spectral resolution of 2.4 cm^−1^. The laser spot diameter at the sample was 105 µm, obtained by using a 20 × Olympus objective (N.A. 0.25, working distance 8.8 mm). Spectra collection was performed by averaging 3 accumulations of 10 s CCD exposure each (30 s in total), and with a laser power at the sample of 120 mW (30% of the maximum laser output). For pure metabolites (i.e. bilirubin, hypoxanthine and xanthine), a laser power at the sample of 40 mW (10% of the maximum laser output) was used for xanthine and bilirubin, and 80 mW (20% of the maximum laser output) for hypoxanthine. For bilirubin, a single accumulation with a 30-s CCD exposure was used. Using these experimental conditions, no substrate photo-degradation was reported. To check the spectrometer wavelength calibration, paracetamol was used as a standard reference sample during every measurement session. To check for measurement repeatability, 5 aliquots for each methanol extract (i.e. for each faecal sample) were measured and compared (see Supplementary material, Fig S4): since in all cases the spectra from the same extract were identical, only 1 spectrum/extract was considered for data analysis.

### SERS data preprocessing and analysis

Spectra have been entirely processed using the R environment for data analysis [17]—version 4.1.0 (2021–05-18). In particular, the package hyperSpec [18] was used for data import and visualization. The preprocessing steps included (i) Raman shift range selection (300–1800 cm^−1^), (ii) baseline correction (package baseline [19], method “als”, lambda parameter = 4) and (iii) vector normalization. Examples of baselines are shown in Fig. S3 of the Supplementary information. Principal component analysis (PCA) was performed using the *prcomp* function, centering but not scaling data. The cumulative proportion of explained variance for the first 19 principal components of the dataset is available as Supplementary information (Fig. S5). The Welch’s unequal variances *t* test with correction for false discovery rate for the scores of the first principal component was performed by using the *pairwise.t.test* function (p.adjust.method = “BH”, pool.sd = FALSE). Spearman’s correlation coefficients between scores of the first principal component and operational taxonomic unit (OTU) relative abundances were computed by using the *cor.test* function, to measure the strength of association between these two variables [20]. For each correlation coefficient, the chance that the correlation is due to chance was estimated by calculating the *p* value (retuned by the *cor.test* function as well). The *p* values obtained were corrected by estimating the false discovery rate (FDR) by using the *p.adjust* function, according to the Benjamini–Hochberg method [21]. All figures were prepared using the R environment for data analysis [17].

### Genomic analysis 

Library preparation and sequencing were performed at the DNA sequencing facility of the Department of Life Sciences of the University of Trieste [22]. Genomic DNA was extracted using the E.Z.N.A® Stool DNA kit (Omega Bio-Tek) following the manufacturer’s instructions. DNA quality and quantity were assessed with a NanoDrop 2000 Spectrophotometer (Thermo Fisher Scientific). An extraction blank was performed as a control to monitor for contamination of environmental bacteria DNA. The extracted DNA was used as a template for the amplification of the V4 hypervariable region of the 16S rRNA by PCR primers 515F/806R [23]. Primers were tailed with two different GC-rich sequences enabling barcoding in a second amplification. For each sample, three technical replicates were performed in 20 µL of volume reaction containing 10 µL AccuStartII PCR ToughMix 2X (Quanta Bio), 1 µL EvaGreen™ 20X (Biotium), 0.8 µL 515F (10 µM- 5′ tailed CAGGACCAGGGTACGGTG), 0.8 µL 806R (10 µM- 5′ tailed with CGCAGAGAGGCTCCGTG-) and 50 ng of DNA template. The amplification was performed in a CFX 96™ PCR System (Bio-Rad) with a real-time limited number of cycles (94 °C for 20 s, 55 °C for 20 s, 72 °C for 60 s). A second PCR amplification (outer PCR) is required to label each sample uniquely and was performed using a forward primer composed of the “A” adaptor, a sample-specific 10-bp barcode and tail 1 of the primary PCR primers, and a reverse primer composed of the P1 adaptor sequence and tail 2. The reactions were performed in 25 µL volume containing 12.5 µL AccuStartII PCR ToughMix 2X (Quanta Bio), 1.25 µL EvaGreen™ 20X (Biotium), 1.5 μL barcoded primer F&R (10 µM), 1 μL of the first PCR product (pool of the three technical replicates) with the following conditions: 8 cycles of 94 °C for 10 s, 60 °C for 10 s, 65 °C for 30 s and a final extension of 65 °C for 2 min. All the amplicons were checked for their quality and size by agarose gel electrophoresis, purified by Mag-Bind®TotalPure NGS (Omega Bio-Tek), quantified with the Qubit Fluorometer (Thermo Fisher Scientific) and pooled together in equimolar amounts. The library was finally checked by agarose gel electrophoresis and quantified in the Qubit Fluorometer. For sequencing, the library was first subjected to emulsion PCR on the Ion OneTouch™ 2 system using the Ion PGM™ Template Hi-Q OT2 View according to the manufacturer’s instructions. Then ion sphere particles (ISP) were enriched using the E/S module. Resultant live ISPs were loaded and sequenced on an Ion 316 chip in the Ion Torrent PGM System (all ION instruments and reagents are from Life Technologies).

### Genomic data preprocessing and analysis

The CLC Microbial Genomics Module as a part of the CLC Genomics Workbench 20.0 (QIAGEN Digital Insights, Aarhus, Denmark) was used to analyse alpha and beta diversity, and the composition of the bacterial community [22]. Raw sequencing reads were imported into the CLC environment, and subjected to quality control, primer and adapter sequence removal and minimum size cut-off of 150 bp. The OTUs were picked by mapping sequences against the SILVA 16S v132 97% database [24] at the same identity percentage to observe OTU at the species level. Next, the OTUs were aligned using multiple sequence comparison by log-expectation and used to construct a “maximum likelihood phylogenetic tree” followed by alpha and beta diversity analyses. We estimated the effect size and significance on beta diversity for grouping variables with PERMANOVA [25]. PERMANOVA is an acronym for “permutational multivariate analysis of variance”, and it is a semi-parametric multivariate statistical test used to compare groups by testing the null hypothesis that the centroids and dispersion of the groups as defined by a distance measure (in our case the Bray–Curtis dissimilarity) are the same for all groups. PERMANOVA applied to our OTU dataset returned pseudo *f*-statistic values [25] and *p* values (Bonferroni corrected) [26, 27]. For a detailed description of the meaning of the pseudo *f*-statistic (or pseudo *f*-ratio), see [25]. Differential abundance analysis [28] was performed by modelling each OTU as a separate generalized linear model (GLM), where it is assumed that abundances follow a negative binomial distribution. The Wald test was used to determine the significance of group pairs.

## Results and discussion

**Fig. 1:**
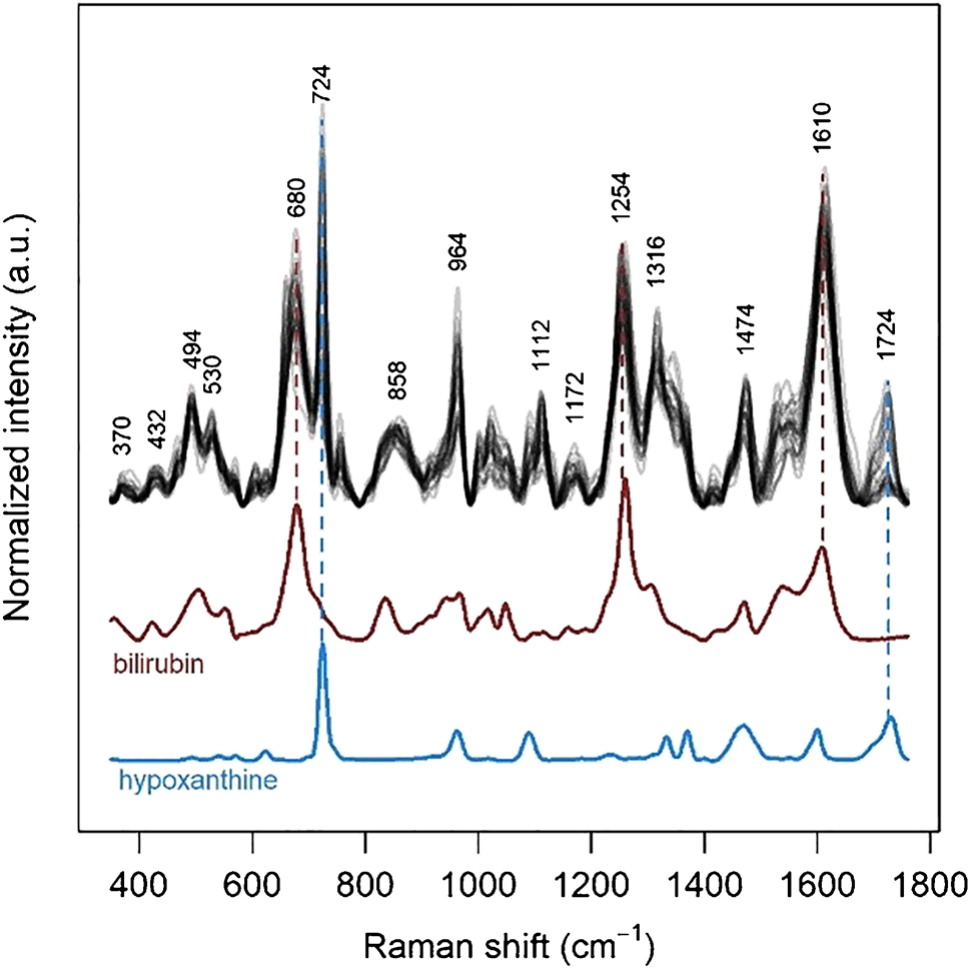
All 27 SERS spectra of the dataset (normalized, overlaid). SERS spectra of bilirubin (dark red) and hypoxanthine (light blue) are reported for comparison. Excitation wavelength 785 nm, AuNP used as SERS substrate

SERS spectra can be readily observed from methanol faecal extracts upon mixing with Au nanoparticle dispersions (Fig. 1). Extraction with methanol is widely used in faecal metabolomics [29], and the protocol used in this study, involving the extraction with methanol and the addition to an aqueous dispersion of Au nanoparticles, has been optimized to maximize the repeatability of SERS spectra. Although normal Raman spectra of faecal samples were reported in literature [30], to the best of our knowledge, these are the first SERS spectra obtained from faecal samples. A detailed comparison between our SERS data and the normal Raman data in literature is difficult, since normal Raman bands were not labelled, and the wavenumber axis labelling in the figures of that paper does not allow for a precise estimate of the Raman shifts of bands maxima. However, the two spectral profiles appear to be very different, with no similar bands. While the SERS data present some variability, all spectra share some common features (Fig. 1). The bands at 722 and 1724 cm^−1^, present with variable intensity in all spectra, are attributed to hypoxanthine on the basis of a direct comparison with the SERS spectrum of this metabolite (Fig. 1).

**Fig. 2:**
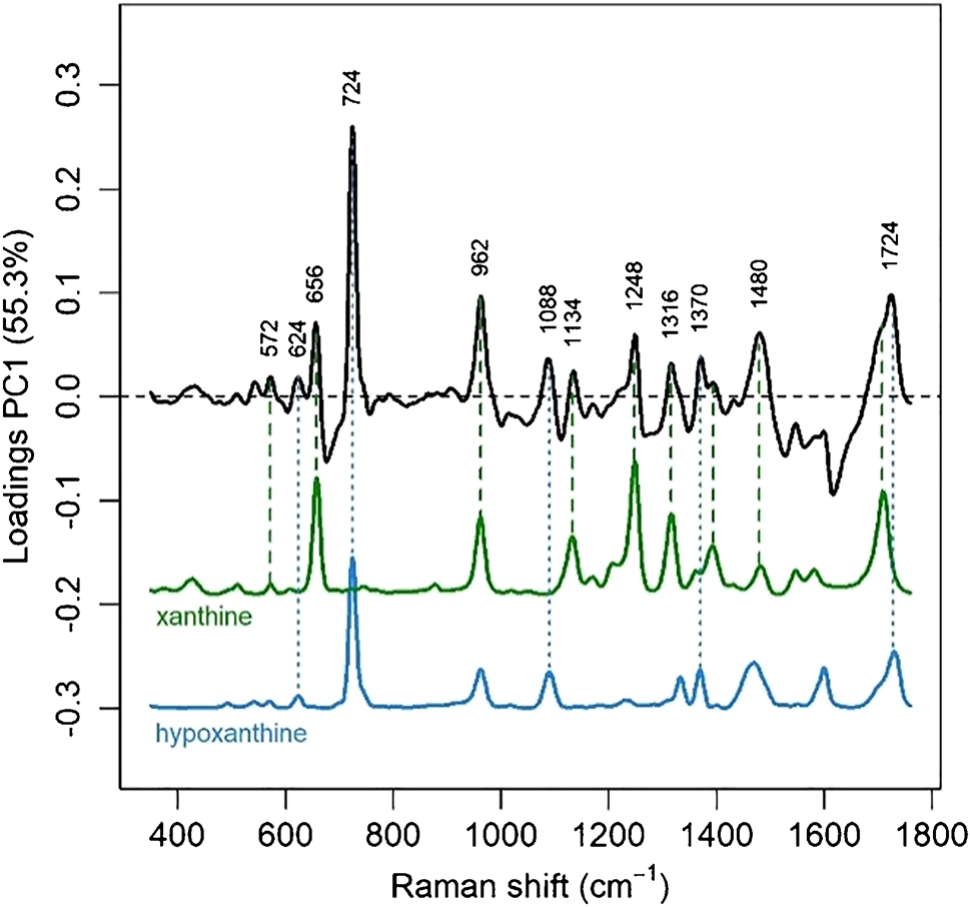
Loadings of the first principal component of the SERS dataset (black), showing which spectral features are responsible for most of the variance. SERS spectra of xanthine (green) and hypoxanthine (light blue) are reported for comparison. Excitation wavelength 785 nm, AuNP used as SERS substrate

Most of the other bands common to all spectra in the dataset appear to be related to a bilirubin-like species. These bands are consistent with bilirubin SERS spectra reported by other authors upon 785-nm excitation [31, 32]. A better and more complete description of the SERS dataset and its variance can be achieved by performing an exploratory analysis such as principal component analysis (PCA). The first principal component of the spectral dataset explains more than half of the spectral variance (55.3%). The second and third principal components explain only 14.9% and 10.1% of the variance, respectively, much less than the first one (Fig. S5 in the Supplementary). The loadings of the first principal component (Fig. 2) show that most of the variability in the SERS spectra of the dataset is due to hypoxanthine bands as well as to other bands which can be attributed on the basis of a direct comparison to xanthine, another purine metabolite. Different from xanthine and hypoxanthine, the bands of bilirubin-like species do not play a major role in the spectral variability of the dataset (Fig. 2), and they only appear in the second principal component (the loadings of the first six principal components are shown in Supplementary material, Fig. S6).

While bilirubin and bilirubin-related species, as products of the heme catabolism, are expected to be found in faeces [33], xanthine oxidase converts hypoxanthine to xanthine and then to uric acid, which is predominantly excreted with urine [34, 35]. On the other hand, around 60% of faecal mass consists of bacteria [36], and bands due to hypoxanthine and xanthine, along with those due to other purine metabolites, have been reported in SERS spectra of several bacteria [37–40], corroborating our interpretation of the bands observed from faecal extracts. Recently, Scott Lee et al. reported hypoxanthine and other purines in faeces of mice, whereas no purines could be detected in germ-free mice, suggesting that these faecal metabolites are indeed produced by bacteria [41]. These evidences suggest that the hypoxanthine and xanthine bands observed in the SERS spectra of faecal extracts reported in this paper are not metabolic products of the host, but metabolites due to the bacterial component of faeces.

Convincing evidence from previous SERS studies [39] indicates that purines due to the metabolic degradation of nucleic acids and nucleotides are secreted by bacteria into extracellular regions, where they can interact with the metallic SERS substrates. Thus, the xanthine and hypoxanthine bands observed in SERS spectra of faecal extracts are likely due to those purine metabolites secreted from faecal bacteria into the solvent. Although a recent study shows that methanol might cause bacterial cell lysis after hours of incubation [42], this is not the case for the few minutes involved in sample preparation for SERS measurements, supporting the hypothesis that the metabolites detected are not due to the content released by lysed cells.

As expected, mass spectrometry data from previous SERS studies on bacteria also showed that many other types of metabolites were present in high concentrations in the bacteria supernatant [39]. Nevertheless, only bands due to purines were observed in SERS spectra, where the affinity for the metal surface selects which analytes are detected and which are not [39]. Heterocyclic aromatic molecules such as purines strongly interact with Au and Ag surfaces, yielding intense SERS spectra even when a large number of other compounds are present. Despite the presence of thousands of other metabolites, purines such as uric acid and hypoxanthine, for instance, dominate the SERS spectra of many biological fluids, such as blood serum, plasma and tears [43]. Thus, an analogous effect is probably occurring also in the case of SERS spectra of faecal extracts, which are known to contain hundreds of metabolites [44, 45].

**Fig. 3:**
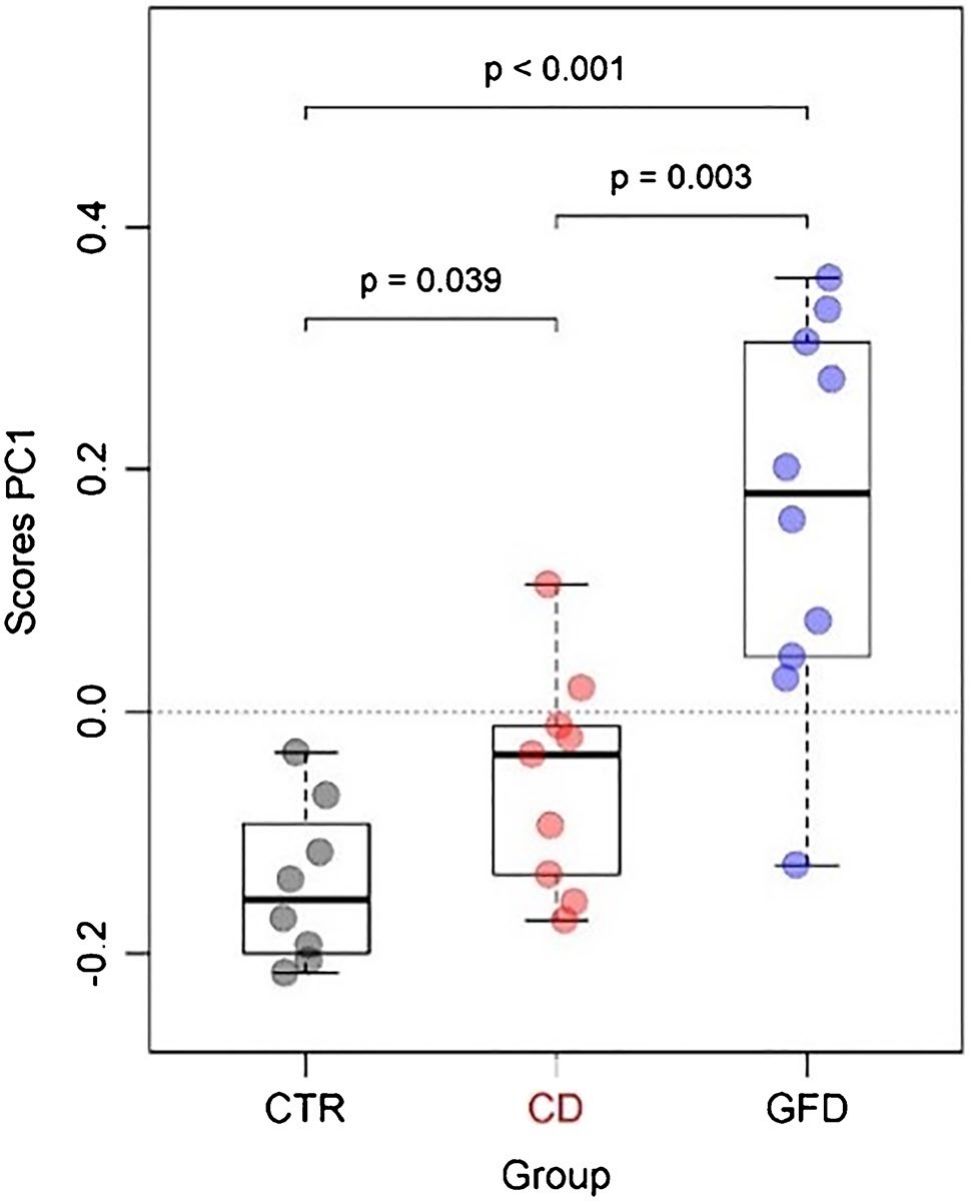
Scores of the first principal component of SERS dataset split by group (controls, CTR; coeliac disease, CD; gluten-free diet, GFD). The *p* value was obtained by a pairwise *t* test (adjusted for false discovery rate with the Benjamini–Hochberg method) with no assumption of equal variances

The scores of the first principal component of the SERS dataset seem to vary among the different groups studied (Fig. 3). Interestingly, the gluten-free diet seems to have a major effect on the spectra, as inferred from the scores, which clearly differentiate the GFD from the other groups. When directly comparing the CD and GFD groups, where the gluten-free diet is the main variable, the PC1 scores of the spectra from samples of the coeliac patients following a gluten-free diet are significantly higher than those of coeliac patients in gluten-containing diet, implying that the bands of hypoxanthine and xanthine, observed as positive loadings in Fig. 2, are more intense in the GFD group.

**Fig. 4:**
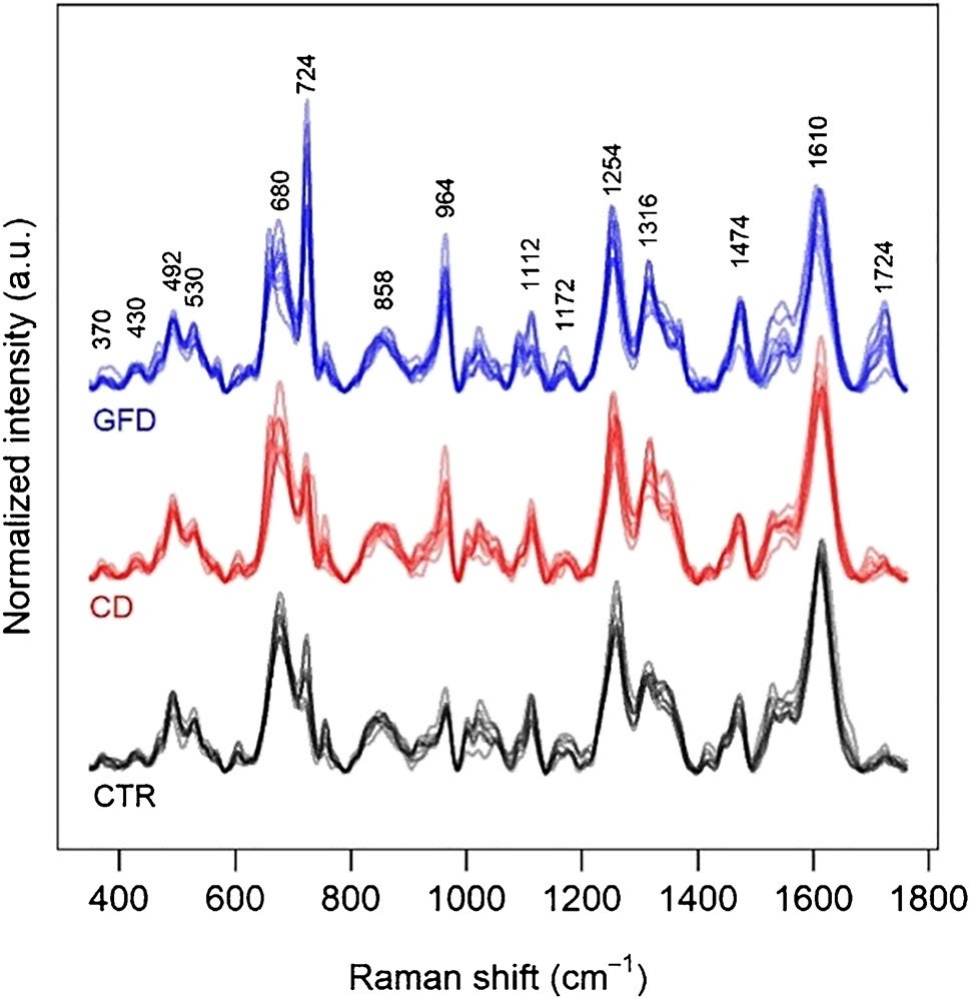
Overlaid SERS spectra of the faecal samples for 3 different groups (controls, CTR; coeliac disease, CD; gluten-free diet, GFD). Excitation wavelength 785 nm, AuNP used as SERS substrate

**Fig. 5:**
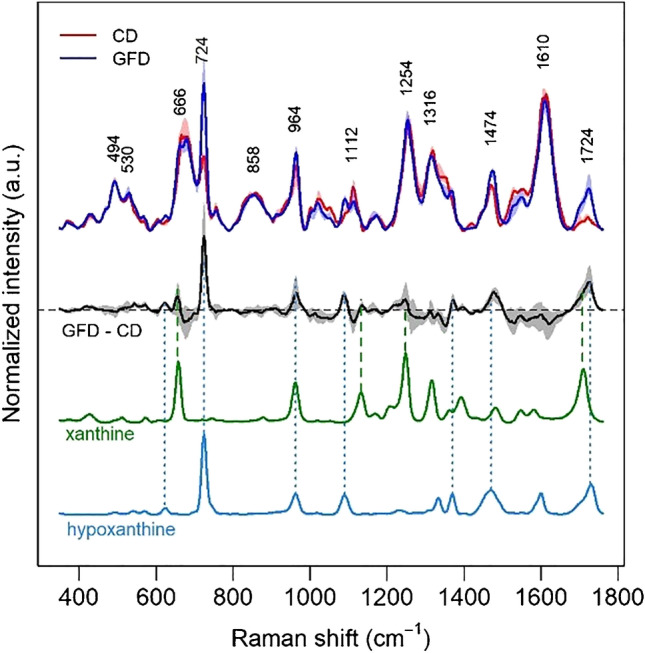
Comparison between medians and interquartiles (shaded areas) of the intensity for the SERS spectra of the coeliac disease (CD, red) and gluten-free diet (GFD, blue) groups, together with the median and interquartile of all the difference spectra (black). SERS spectra of xanthine (green) and hypoxanthine (light blue) acquired in the same conditions are reported for comparison. Excitation wavelength 785 nm, AuNP used as SERS substrate

This feature can be appreciated also by simply looking at the spectral dataset split in the three groups (Fig. 4), where the hypoxanthine band at 722 cm^−1^ is more intense in the GFD group. The median of the spectral differences between the spectra from the samples of the GFD and CD groups (Fig. 5) confirms these features, highlighting a higher relative intensity also of a xanthine band, consistently with what was suggested by the loadings graph in Fig. 2. On the other hand, Fig. 4, in agreement with the PC1 scores of Fig. 2, shows that spectral differences between the spectra of the CD and CTR groups are much smaller than those between the spectra of the CD and GFD groups. A figure reporting a direct comparison between spectra of the CTR and CD spectrum, analogous to Fig. 5, is shown in the Supplementary material (Fig. S7).

To check if these spectral differences are reflecting a different bacterial composition of the faecal samples of the three groups, a microbiota analysis of all samples was performed by sequencing V4 PCR amplicons from the ribosomal 16S RNA genes. Amplicon sequencing produced a total of 3,556,159 reads with an average of 107,762 ± 22,119 reads per sample. Raw sequences (reads) were quality filtered, then trimmed from primers and adapters. The remaining sequences (2,377,022 reads) of 250 bp in length were reference-based clustered against the SILVA 16S v132 database with a 97% sequence similarity accounting for 2915 reference-based OTUs and 1128 de novo OTUs from the 27 assayed samples. The mean number of reads in OTUs was 62,658 ± 7549 for the control, 63,309 ± 11,501 for CD and 72,312 ± 17,413 for GFD samples. Rarefaction curves calculated for total OTU abundance reached the plateau indicating that sequencing was adequate to analyse the majority of phylotypes in all the samples (Supplementary fig. S8).

**Fig. 6:**
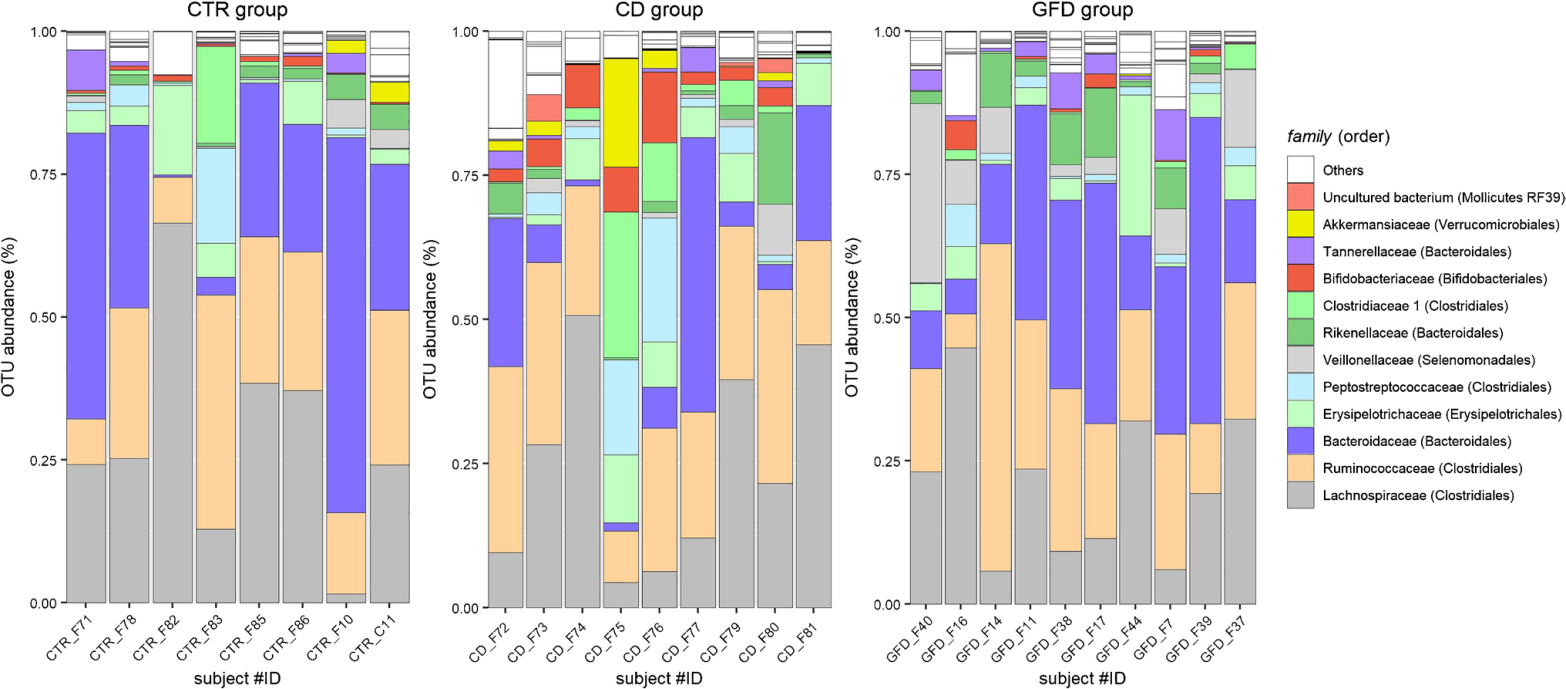
Composition of bacterial communities at the family level in the 3 different groups. CD: coeliac disease, CTR: control, GFD: gluten-free diet

The analysis of the results at the “family” taxonomic level (Fig. 6) showed that *Lachnospiraceae*, *Ruminococcaceae* (order Clostridiales) and *Bacteroidaceae* (order Bacteroidales) are the most represented families of bacteria in the faecal samples (on average, 75% of the total reads), in line with the predominance of the Firmicutes and Bacteroidetes phyla previously reported in studies on gut microbiome composition [46, 47]. A total of 79 genera and 26 prokaryotic families are recognized in the stool microbiota. A certain variability is observed for each group, the GFD group being the most homogeneous.

A PERMANOVA (Table 2) suggests that the three groups present significant differences. As in the case of SERS spectra, the GFD seems to have a major effect on the microbiota. We have hypothesized that the diet is responsible for the spectral and microbiological differences observed between the CD and GFD groups. However, this is a descriptive case study performed on a small number of subjects with ages not perfectly matching in the three groups, so it cannot be excluded that age variations between CD and GFD groups might concur as well to the differences observed. An impact on gut microbiota by GFD has been reported in a few studies [48, 49], although with no clear consensus on the results. To better understand which bacteria are responsible for this difference in our dataset, a differential abundance analysis has been performed (Table S9 of the Supplementary Information), showing that three families (i.e. an uncultured bacterium from the order of the Mollicutes RF39—phylum Tenericutes, *Akkermansiaceae*—phylum Verrucomicrobia and *Clostridiaceae* 1—phylum Firmicutes) are significantly less present in the GFD group than in the CD group. Comparison at the genus level highlighted the Rikenellaceae RC9 gut group (order Bacteroidales), uncultured bacterium (order Mollicutes RF39) and Akkermansia were statistically less represented in GFD CD patients than in CD (all with a *p* < 0.001, Bonferroni corrected). On the contrary, *Escherichia-Shigella* is more represented in the former group, although a major difference is shown in the GFD-vs-CTRL comparison. Several studies reporting the effects of a GFD on the microbiota of coeliac patients were unfortunately based on a variety of methods, kind of samples (usually deriving from adults) and even different statistical approaches for data analysis [48, 49], making a direct comparison with our results problematic.

**Table 2:** Results from PERMANOVA on Bray–Curtis distances for the data obtained from the 16S rRNA gene analysis

Groups		Pseudo *f*-statistics	*p* value	
CTR, CD, GFD		1.367	0.040	
Group 1	Group 2	Pseudo *f*-statistics	*p* value	*p* value (Bonferroni)
CD	CTR	1.231	0.207	0.622
CD	GFD	1.664	0.015	0.046
CTR	GFD	1.187	0.203	0.609

**Table 3:** Spearman’s correlation coefficients (and the relative FDR *p* values) between OTU’s relative abundances and PC1 scores of the SERS dataset for the bacterial families found by the differential abundance analysis

Name	Correlation coefficient	FDR *p* value
Uncultured bacterium (Mollicutes)	− 0.03	0.98
*Akkermansiaceae*	− 0.29	0.83
*Clostridiaceae 1*	− 0.13	0.98

Spearman’s correlation coefficients between the OTU’s relative abundances and the PC1 scores of the SERS dataset for each sample (Table 3) clearly indicate that there is no correlation between SERS data and the occurrence of these bacterial families (nor for other families, data not shown). The absence of any correlation suggests that SERS spectra reflect bacterial metabolism rather than bacterial composition of faecal samples. While further studies on a larger sample size planned with a careful study design will be able to corroborate our hypothesis, for the moment, it might be reasonable to assume that the products of the metabolism of the same bacteria are different depending on the nutrients available from dietary intake. In other words, the SERS spectrum of a bacterium is not a unique fingerprint of that species, but rather a probe of its mutable metabolic state. This hypothesis is consistent with the results reported by Weiss et al. on SERS spectra of different bacteria upon varying metabolic conditions [38]. This could explain why bacterial species present in similar amounts in faecal samples of both the CD and GFD groups might still show different metabolic profiles, which would translate into a different composition of faecal extracts and thus in different SERS spectra.

From this perspective, the metabolic information conveyed by SERS data is complementary to that of the bacterial composition as given by the 16S rRNA gene, allowing us to take a look at the faecal samples from another viewpoint. The biochemical reason behind the increased production of hypoxanthine and xanthine by the microbiota of the GFD group with respect to other groups remains unclear. Some SERS studies on bacteria reported an increase in purine production upon starvation or physiological stress [37–39, 50], but to the best of our knowledge, such metabolic changes have never been reported for the microbiota of subjects following a gluten-free diet. As a further layer of complexity making the biochemical interpretation difficult, bacterial metabolism might also depend on the interaction between different species, and a simple reductionist approach might be inadequate to represent the complexity of the microbial ecosystem of the gut [51–53].

In the present study, such metabolic information appears to be limited to just two metabolites, hypoxanthine and xanthine, and cannot compete with the wealth of information provided by traditional metabolomics approaches. Since the information present in SERS spectra depends on the interaction between the analytes and the nanostructured metal substrates, the number and type of metabolites detected by a label-free SERS approach in faecal samples might change if other methods (e.g. other solvents or sampling protocols) or substrates (e.g. Ag surfaces or Au surfaces with different characteristics) are used [54], possibly modifying or expanding the metabolic information available. The use of solvents with a different polarity, for instance, might change the nature and/or concentration of the metabolites present in the faecal extracts. The use of more hydrophobic, or perhaps positively charged, SERS substrates might increase the affinity of the nanostructured metal surface for bacterial metabolites other than hypoxanthine and xanthine.

On the other hand, by comparing SERS on deposited AuNP and MS data obtained from the same bacteria supernatant fluid, Premasiri et al. clearly showed how these two techniques have very different sensibilities toward different metabolites, and must be thus considered as complementary [39]. Moreover, SERS analysis is much faster and less expensive than methods based on HPLC and NMR or MS, and might be used for a quick characterization of faecal samples. For instance, the SERS approach described in this paper, if validated on larger datasets in future studies, could be a possible tool for the assessment of the compliance to the gluten-free diet, which has been reported as a major practical clinical problem in CD patient follow-up [55, 56] or as an indicator of the restoration of normal villous architecture and mucous barrier integrity [41].

## Conclusions

SERS spectra can be consistently obtained from faecal samples upon addition of AuNP to their methanol extracts. The bands observed in these spectra can be attributed to bilirubin-like species as well as to purine metabolites (i.e. xanthine and hypoxanthine) that are most likely secreted from the bacteria present in the gut. Significant differences concerning the bands of these two xanthines are observed between the spectra of coeliac patients and those of coeliac patients following a GFD, suggesting that the purine metabolism of the gut bacteria of the two groups is different. Moreover, spectral differences do not correlate with differences in bacterial composition (at the family level) as derived from a genomic analysis, indicating that SERS spectra are, presumably, not reflecting the bacterial composition of faecal samples, but rather the metabolic state of the bacterial community. In this sense, SERS appears to be complementary to 16S rRNA sequencing analysis for the characterization of faecal samples. The results reported suggest that SERS could be used as a fast and relatively inexpensive technique to assess the compliance of patients to the GFD, or perhaps the degree of mucosal recovery. A limitation of this preliminary study is the small sample size, so that the results reported need to be confirmed by further studies on a larger sample size, which will also allow us to evaluate the accuracy of this approach to assess GFD compliance.

## Supplementary Information

Below is the link to the electronic supplementary material.

Supplementary file1 (DOCX 1.25 MB)

## Data Availability

The dataset consisting of all spectra is available for download on Zenodo (zenodo.org), https://doi.org/10.5281/zenodo.5947010.

## Code availability

The R code used for data pre-processing, analysis and visualization is available for download on Zenodo (zenodo.org), https://doi.org/10.5281/zenodo.5947010.
