# Supplementary material for: Spectroscopic investigation of faeces with surface-enhanced Raman scattering: a case study with coeliac patients on gluten-free diet
Source: Anal Bioanal Chem. 2022 Mar 8;414(11):3517–27. doi: 10.1007/s00216-022-03975-y (PMC9018641; doi:10.1007/s00216-022-03975-y)
Supplement: Supplementary file 1 — Supplementary file1 (DOCX 1.25 MB) [file 216_2022_3975_MOESM1_ESM.docx]

Supplementary Information for:

Spectroscopic investigation of faeces with surface-enhanced Raman scattering: a case study with coeliac patients on gluten-free diet

Stefano Fornasaro^‡a^, Alessandro Esposito^‡a^, Fiorella Florian^b^, Alberto Pallavicini^b^, Luigina De Leo^c^, Tarcisio Not^c^, Cristina Lagatolla^b^, Marica Mezzarobba^b^, Alessia di Silvestre^a^, Valter Sergo^a^, and Alois Bonifacio*^a^.

^a^ Raman Spectroscopy Lab, Department of Engineering and Architecture, University of Trieste, P.le Europa 1, 34100 Trieste, Italy; ^b^ Department of Life Sciences, University of Trieste, Via Edoardo Weiss 2, 34128 Trieste (TS), Italy; ^c^ Institute for Maternal Child Health-IRCCS “Burlo Garofolo” Trieste, via dell’Istria 65/1, 34100 Trieste, Italy

**Figures**


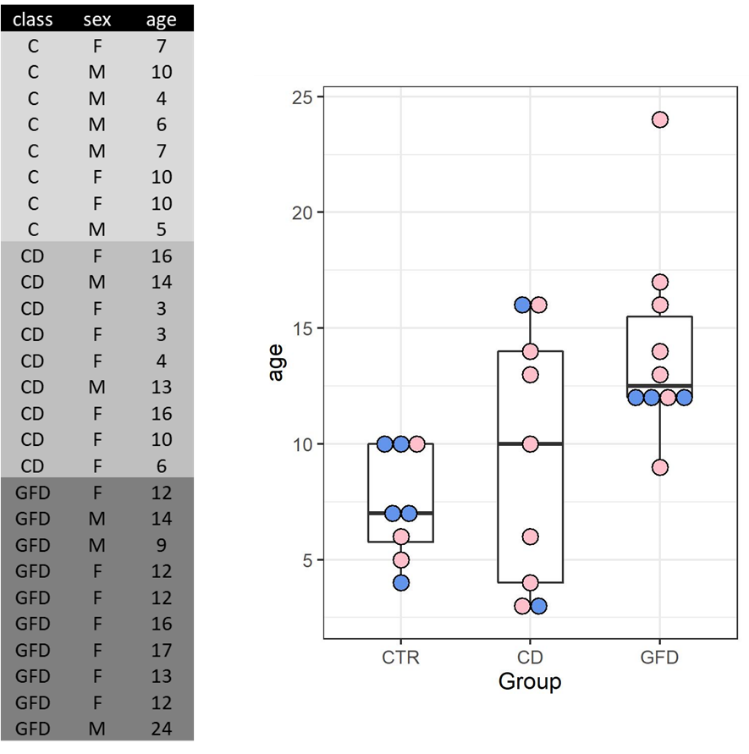


**Figure S1**: (left) characteristics (age, sex) of the subjects participating to the study. In the boxplot (right) blue dot = male, pink dot = female.


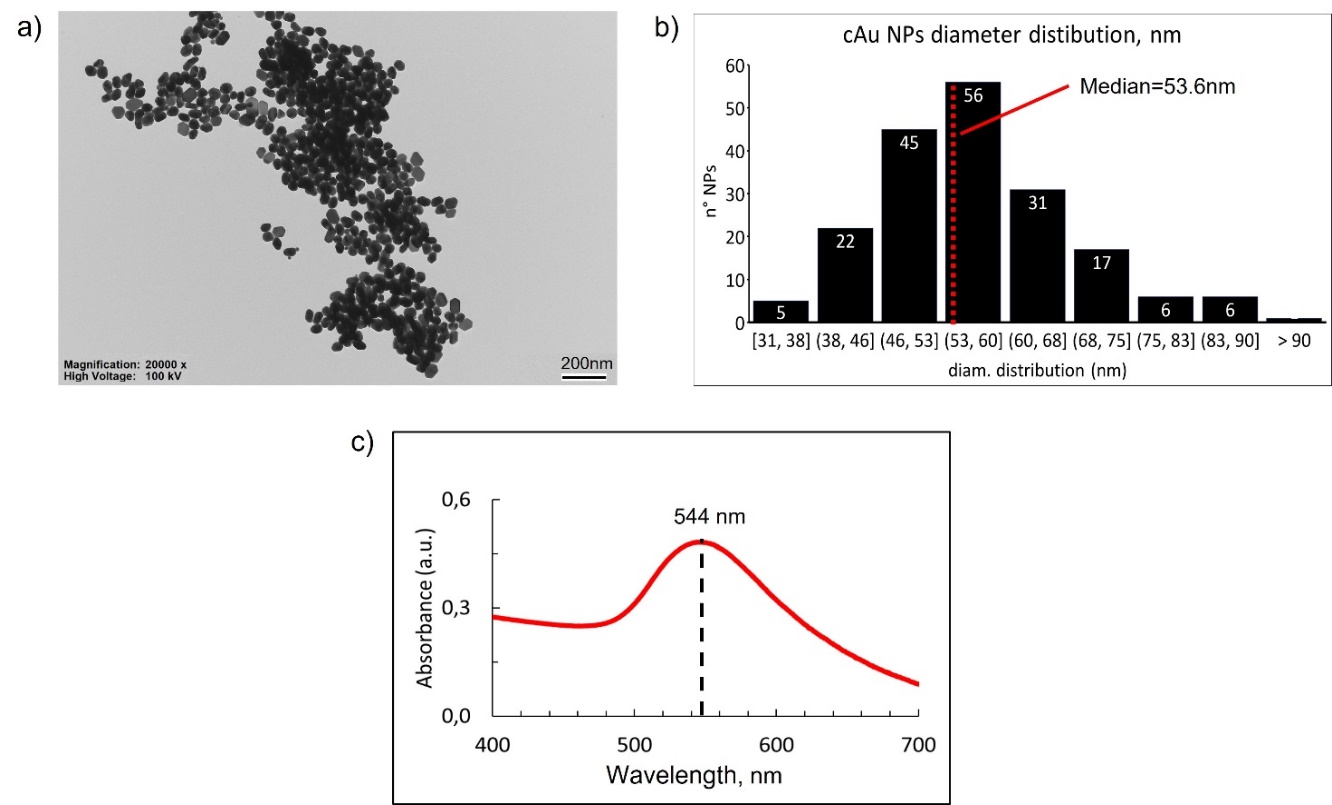


**Figure S2*.*** Characterization of AuNP: a) TEM image of AuNP, b) size distribution of AuNP as derived from TEM, c) Visible extinction spectrum of the colloidal dispersion of AuNP (10-fold diluted).


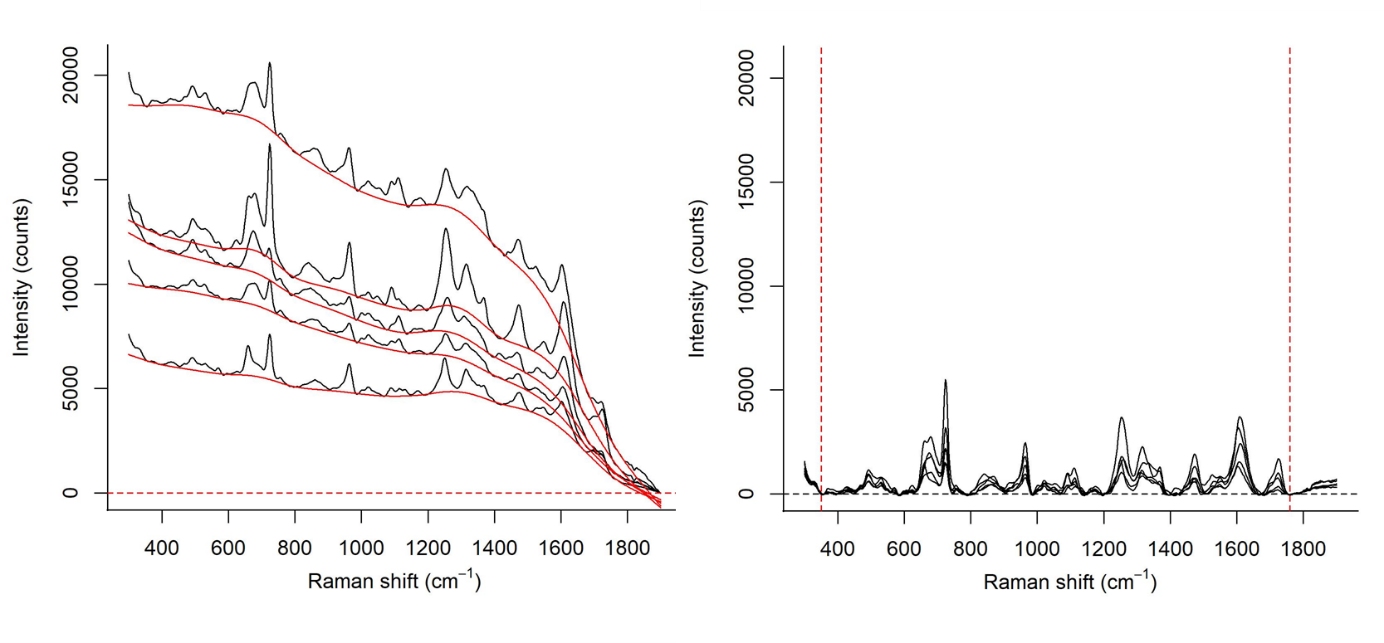


**Figure S3**: Examples of baseline subtraction and spectral range cropping shown for a random selection of 5 spectra from the dataset (see Methods section of the main manuscript for details).


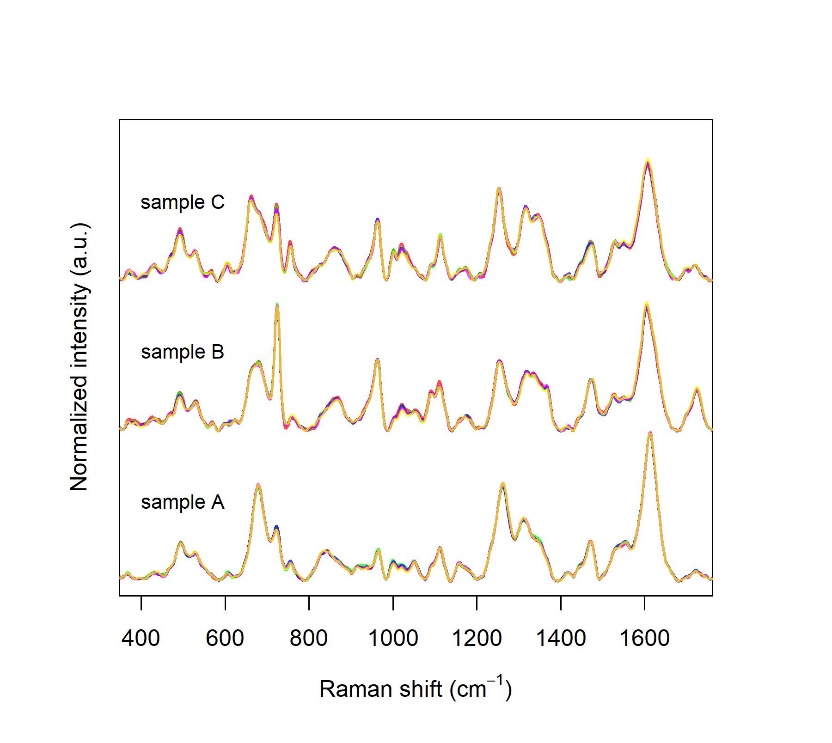


**Figure S4**: replicates of SERS spectra (from 5 aliquots of the faecal extract) for 3 randomly selected samples. Spectra have different colors and are overlaid, showing a high repeatability of SERS measurements. Excitation wavelength 785 nm, AuNP used as SERS substrate.


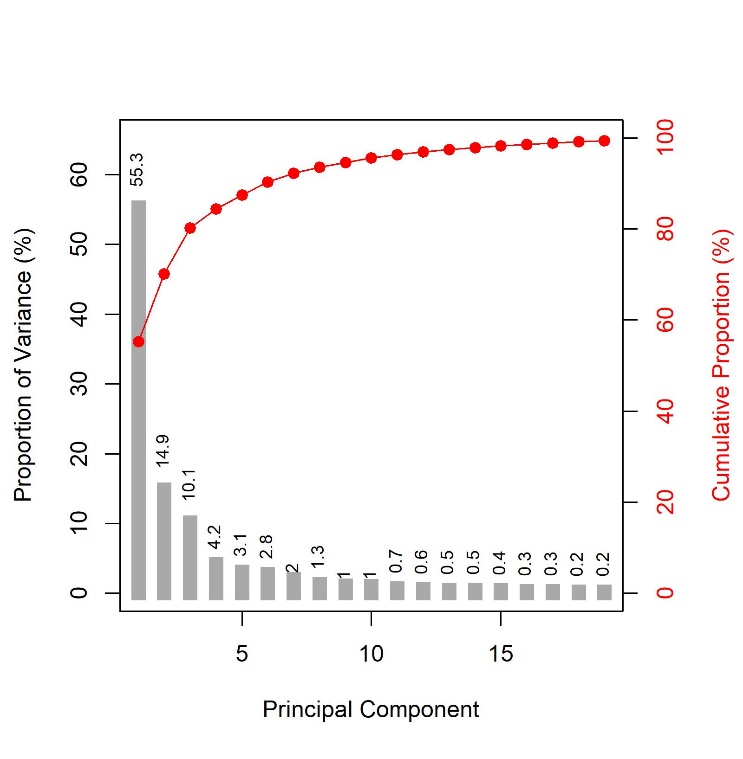


**Figure S5**: Proportion of variance and cumulative proportion of variance explained for the first 19 principal components according to the PCA.


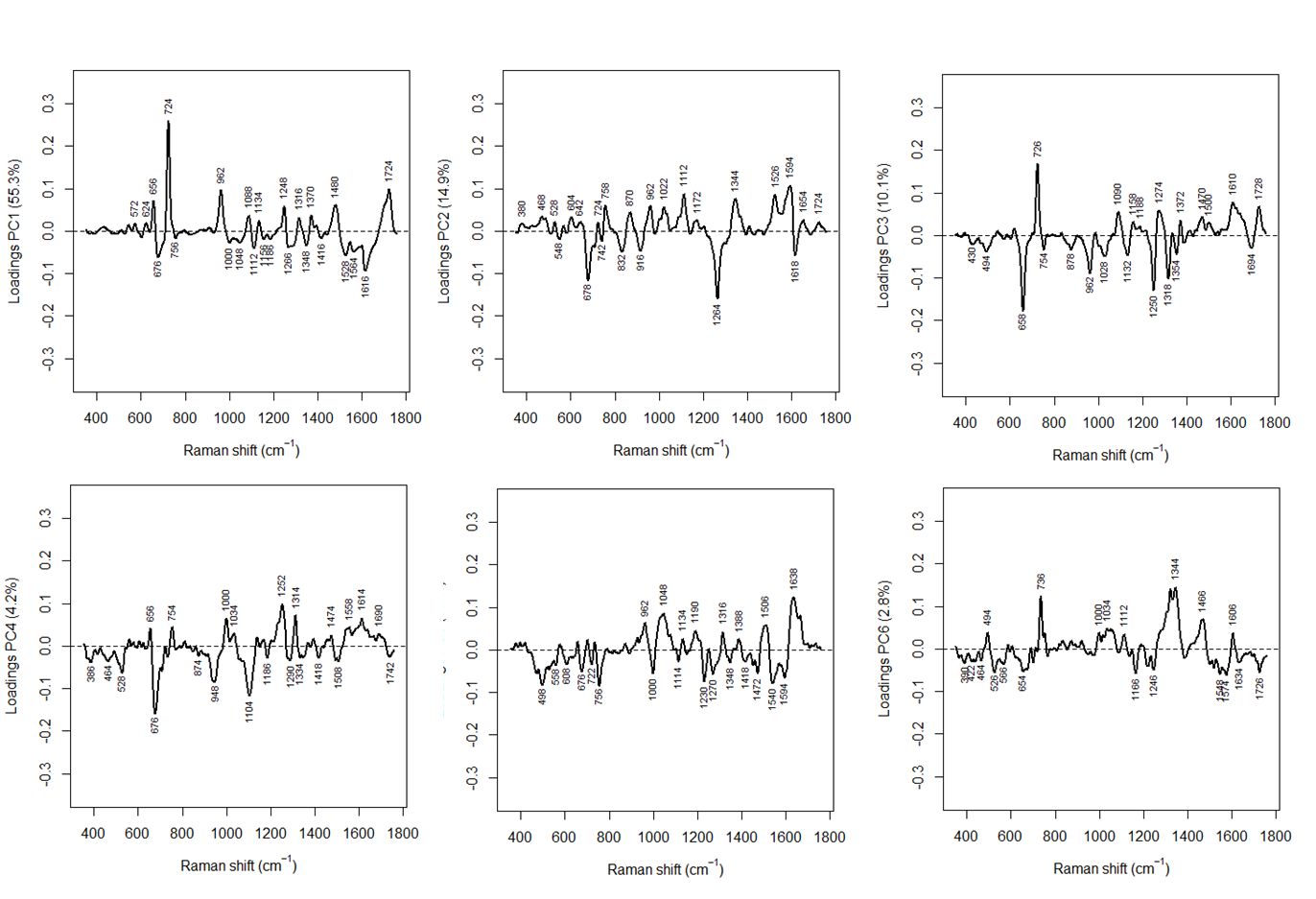


**Figure S6**. Loadings of the first six principal components of the SERS dataset.


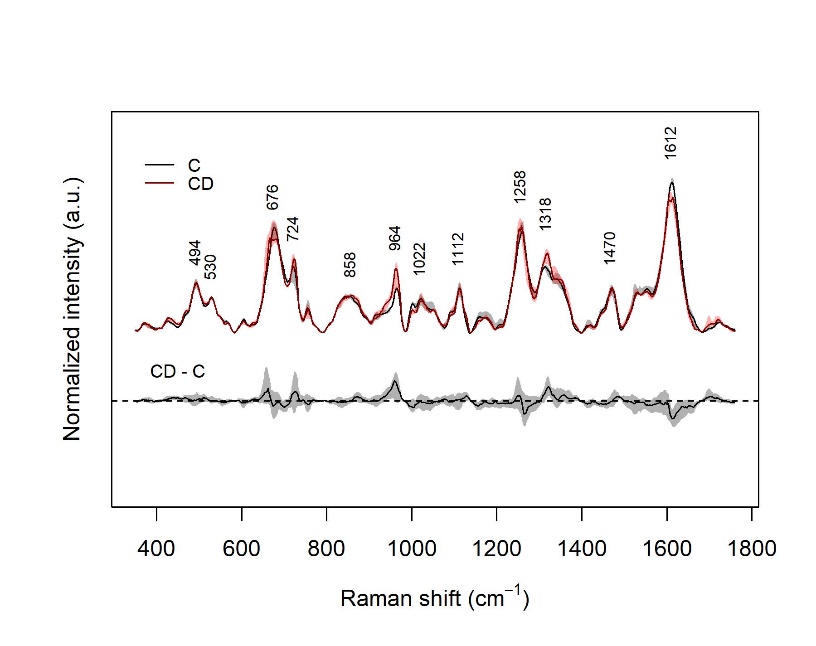


**Figure S7**. Comparison between medians and interquartiles (shaded areas) of the intensity for the SERS spectra of the celiac disease (CD, red) and controls (C, blue) groups, together with the median and interquartile of all the difference spectra (black). Excitation wavelength 785 nm, AuNP used as SERS substrate


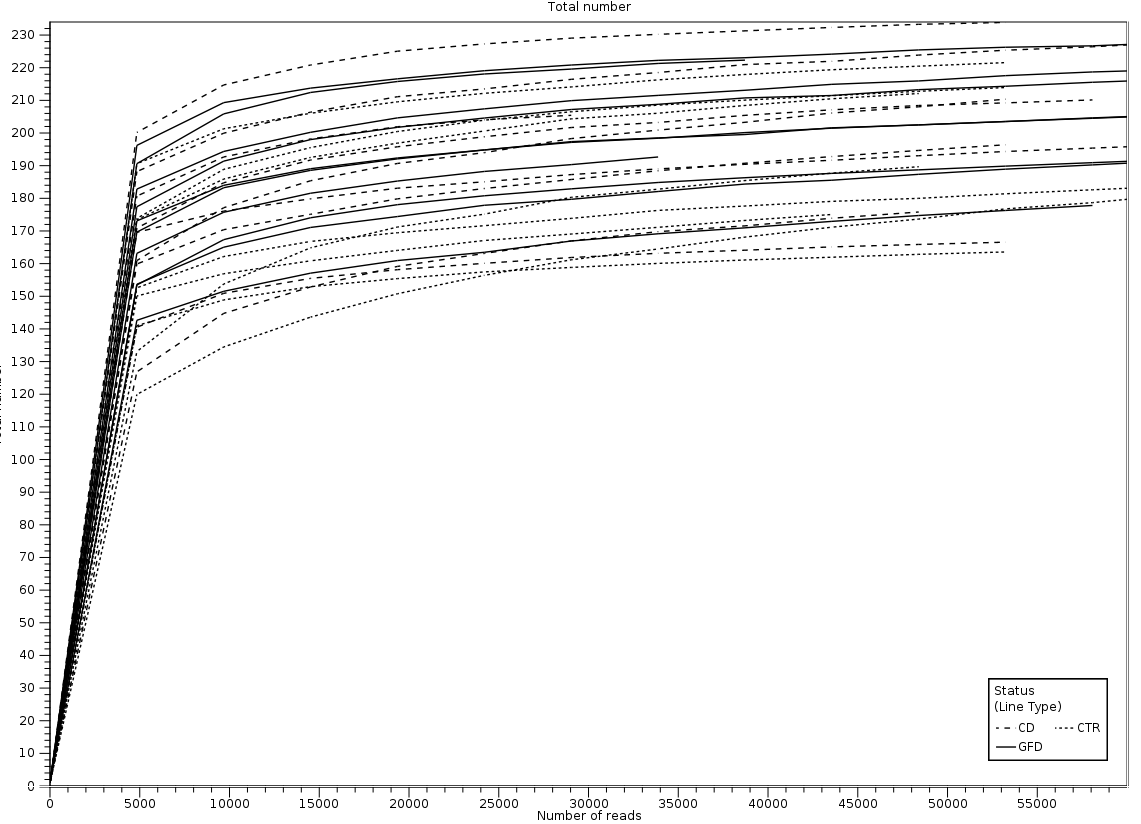


**Figure S8**. Rarefaction curves calculated for total OTUs abundance

| Name | Max group mean | Log2 fold change | Fold change | p-value | FDR p-value |
| --- | --- | --- | --- | --- | --- |
| *Uncultured bacterium*  (order Mollicutes) | 447.67 | -11.33 | -2582.15 | <0.001 | <0.001 |
| *Akkermansiaceae*  (order Verruvomicrobiales) | 1369.44 | -7.42 | -170.97 | <0.001 | <0.001 |
| *Clostridiaceae 1*  (order Clostridiales) | 2315.33 | -2.98 | -7.88 | 0.020 | 0.020 |

**Table S9**. Results from a Differential Abundance Analysis (GFD vs. CD). Only the bacterial families with a Bonferroni-corrected p-value ≤ 0.05 are shown.
